# Veterinarians as a Risk Group for Zoonoses: Exposure, Knowledge and Protective Practices in Finland

**DOI:** 10.1016/j.shaw.2021.10.008

**Published:** 2021-11-09

**Authors:** Paula M. Kinnunen, Alisa Matomäki, Marie Verkola, Annamari Heikinheimo, Olli Vapalahti, Hannimari Kallio-kokko, Anna-Maija Virtala, Pikka Jokelainen

**Affiliations:** 1 Department of Veterinary Biosciences, Faculty of Veterinary Medicine, University of Helsinki, Finland; 2 Division of Health and Social Services, Legality and Licensing, Regional State Administrative Agency of Northern Finland, Finland; 3 Department of Food Hygiene and Environmental Health, Faculty of Veterinary Medicine, University of Helsinki, Finland; 4 Laboratory and Research Division, Microbiology Unit, Finnish Food Authority, Finland; 5 Virology, Medicum, University of Helsinki and HUSLAB, Helsinki University Hospital, Finland; 6 HUS Diagnostic Center, HUSLAB, Clinical Microbiology, University of Helsinki and Helsinki University Hospital, Finland; 7 Infectious Disease Preparedness, Statens Serum Institut, Denmark

**Keywords:** Occupational health, Personal protective equipment, Zoonotic infections

## Abstract

**Background:**

Veterinarians may encounter a variety of zoonotic pathogens in their work.

**Methods:**

We conducted two cross-sectional questionnaire studies among veterinarians in Finland. Participants were recruited during two Annual Veterinary Congresses. In 2009, 306 veterinarians participated in an extensive questionnaire study, and in 2016, 262 veterinarians participated in a more focused study that included two same questions.

**Results:**

In 2009, the majority (90.9%) of the participating veterinarians reported having been occupationally exposed to zoonotic pathogens. Zoonotic infections (15.0%), needle stick incidents (78.8%), bites (85.0%), as well as infected skin lesions (24.2%) were reported. In 2009, 8.2% of the participants fully agreed with the statement “I have good knowledge of zoonoses and their prevention”; in 2016, the proportion was 10.3%. The reported use of protective practices and personal protective equipment in connection with specific veterinary procedures indicated that there was room for improvement, particularly in protection from pathogens that are transmissible via inhalation and mucous membranes.

**Conclusion:**

The results confirm that veterinarians are commonly occupationally exposed to zoonotic pathogens. Education should aim to improve and maintain the knowledge of zoonoses and their prevention. Use of protective practices should be advocated.

## 1 Introduction

Zoonotic pathogens – including bacteria, viruses, parasites and fungi – can be transmitted between animals and humans and may result in subclinical infections or mild to severe, even fatal diseases. Control of zoonoses requires a One Health approach [1]. Worldwide, veterinarians, veterinary students, nurses, and technicians as well as other assisting staff at veterinary workplaces can be exposed to endemic or imported zoonotic pathogens at work [[2], [3], [4], [5]]. The knowledge and actions of everyone in the veterinary work environment, including cleaners, management, and animal owners, are important in ensuring safe workplaces.

Zoonoses are a substantial occupational health risk to veterinarians also in Finland [6]. Based on the probability of transmission and severity of sequelae, *Campylobacter* spp., *Capnocytophaga* *canimorsus*, toxigenic *Escherichia coli*, *Listeria monocytogenes*, *Pasteurella* spp., *Salmonella* spp., methicillin-resistant *Staphylococcus aureus* (MRSA; [7]), *Cryptosporidium* spp., *Toxoplasma* *gondii* [8], and lyssaviruses (including rabies) are considered the main risks to veterinarians in the country [9]. Most of these are endemic and some might be introduced by animal import [10,11]. New threats have recently emerged, for example, livestock-associated MRSA CC398 [7] and Severe Acute Respiratory Syndrome Coronavirus 2 (SARS-CoV-2) [12,13].

Many zoonotic pathogens are transmitted by direct contact or fecal-orally, some through abraded or even intact skin or via bites, some by inhalation or via mucous membranes, and some are vector-borne [5,14]. As many zoonotic health hazards that are relevant for occupational health of veterinarians cannot be eliminated or controlled with engineering solutions (e.g. clinic design), the work processes and protective practices (e.g. hand hygiene and personal protective equipment) are important [14]. In Finland, few local protection and hygiene guidelines have recently become available [15,16].

In recent years, studies worldwide have focused on occupational health and zoonotic infections of veterinarians as well as on their adherence to protective practices (e.g. [[17], [18], [19], [20], [21], [22], [23]]), highlighting the importance of these topics. However, these aspects have not been studied in Finland before the series of research studies this paper belongs to. In this article, we summarize a selection of results from two questionnaire studies. Reported results from the study from 2009 cover self-reported exposure to zoonoses, knowledge of zoonoses, and use of protective practices in connection with several specific veterinary procedures. Furthermore, we compare the self-reported agreement of having good knowledge of zoonoses as well as self-reported hand hygiene practices between the time points of the two studies, 2009 and 2016.

## 2 Materials and methods

We conducted two questionnaire studies among veterinarians authorized to work in Finland. Both studies were cross-sectional and based on convenience samples. The target population was veterinarians working in Finland, and the study populations were the veterinarians attending the Annual Veterinary Congress in 2009 and 2016, respectively. The studies were approved by the Ethics Committee of the Hospital District of Helsinki and Uusimaa (303/13/03/00/09 and HUS/1446/2016). Participation was voluntary, and participants signed an informed consent. It was possible that some same persons participated in both studies. The data were pseudonymized (independently in the two studies) and handled and analyzed coded. The questionnaires are available from the corresponding author upon request.

The first study was an extensive questionnaire study, a part of a large study entity on zoonotic infections of veterinarians, conducted at the Annual Veterinary Congress held in Helsinki, 2009. Of the 1155 congress attendees, 393 (34.0%) participated in the study. The study was mentioned online before the congress, and each attendee received an information sheet. Altogether 306 veterinarians completed an extensive Web-based questionnaire (E-lomake version 3, Eduix Ltd, https://e-lomake.fi/en/) covering demographic and other background information, work environment, animal contacts, exposure to zoonoses, knowledge of zoonoses, and protective practices in connection with a selection of specific veterinary procedures. The skip-pattern questionnaire was available in the official languages, Finnish and Swedish, from 23 October 2009 to 31 January 2010. The questionnaire was technically tested and piloted beforehand by nine people, including four veterinarians, and the questions were edited for clarity. The selected veterinary procedures reflected various animal species as well as potential pathogens and their transmission routes, including direct contact, fecal-oral and percutaneous routes, droplets on mucosal membranes, and inhalation. The questions about protective practices in connection with specific procedures were only answered by those veterinarians who reported performing the procedures. The questions were formulated as “How do you typically protect yourself in connection with performing [procedure] on [animal species]”, and the participants were instructed to choose all the radio buttons applicable (Supplementary Tables 1–12). The question about knowledge was a statement of the knowledge being good, which was not further defined, and the participants selected how much they agreed with the claim. Results of other substudies of the large study entity, focusing on specific pathogens (*T. gondii*, hepatitis E virus, protoparvoviruses, and rodent- and insectivore-borne viruses), have been reported earlier [8,[24], [25], [26]].

The second study was a more limited questionnaire study, a part of a study entity on antimicrobial-resistant bacteria in veterinarians, conducted at the Annual Veterinary Congress held in Helsinki, 2016. Of the 1298 congress attendees, 320 (24.7%) participated in the study. The study was pre-advertised in a local professional journal and on social media. Altogether, 262 veterinarians completed the questionnaire, which was piloted beforehand by 14 veterinarians and edited for clarity. The questionnaire included two same questions as the 2009 study: on knowledge on zoonoses and protective practices in connection with examining wounds. Results of other sub-studies of the study entity, focusing on multidrug-resistant bacteria and infection prevention and control practices of ambulatory veterinarians, have been reported earlier [7,27].

The data were processed in Microsoft Excel, and SPSS (IBM SPSS versions 22 and 25, Armonk, NY, USA) was used for frequency tables and cross-tabulations. To compare proportions, 95% confidence intervals were calculated using Wilson's method [28] with an online calculator (http://epitools.ausvet.com.au/content.php?page=CIProportion). Statistical significance of differences between key proportions was evaluated with z-test (https://epitools.ausvet.com.au/ztesttwo). The *p*-values were corrected for multiple comparisons with the Benjamini and Hochberg method [29] using a false discovery rate (FDR) calculator (https://www.sdmproject.com/utilities/?show=FDR). Statistical significance was considered present at 0.05 level (FDR-corrected *p*-value). For comparisons between 2009 and 2016, the proportions were considered independent because the extent of participation in both studies was expected to be minor.

## 3 Results

### 3.1 Participants, 2009

Table 1 summarizes the background information about the veterinarians who participated in the study in 2009. The participants (N = 306) comprised 15% of the authorized veterinarians in Finland (N = 2026, the Registry of Veterinarians, Finnish Food Authority) and were born between years 1930 and 1986, most during the 1970s. The majority of the participants were female (86.3%, Table 1). Several veterinarians reported an immune system related disease or immunosuppressive medication. The participants did different types of veterinary work; small animal practice was most common. Altogether 80.1% reported doing clinical practice, and 45.6% did mixed practice.

**Table 1:** Background information on veterinarians who participated in the study in Finland in 2009 (N = 306)

	N	%	95% CI
**Gender**			
Female	264	86.3	82.0–89.7
Male	42	13.7	10.3–18.0
**Birth decade**			
1930–1939	3	1.0	0.3–2.8
1940–1949	15	4.9	3.0–7.9
1950–1959	49	16.0	12.3–20.5
1960–1969	86	28.1	23.4–33.4
1970–1979	120	39.2	33.9–44.8
1980–1989	33	10.8	7.8–14.8
**Immune system–related disease**			
Yes	85	27.8	23.1–33.0
No	110	35.9	30.8–41.5
No answer	111	36.3	31.1–41.8
**Immunosuppressive medication**			
Yes	28	9.2	6.4–12.9
No	122	39.9	34.5–45.5
No answer	156	51.0	45.4–56.5
**Work type**∗			
Any clinical practice	245	80.1	75.2–84.2
Small animal practice	215	70.3	64.9–75.1
Production animal practice	145	47.4	41.9–53.0
Equine practice	103	33.7	28.6–39.1
Research	43	14.1	10.6–18.4
Veterinary public health	40	13.1	9.7–17.3
Teaching	39	12.7	9.5–16.9
Administration	33	10.8	7.8–14.8
Other	29	9.5	6.7–13.3
Industry	13	4.2	2.5–7.1
No answer	1	0.3	0.1–1.8
**Working country**			
Only Finland	258	84.3	79.8–88.0
Finland and abroad	41	13.4	10.0–17.7
Only abroad	3	1.0	0.3–2.8
No answer	4	1.3	0.5–3.3

CI, Confidence interval; n, number of participants choosing each option.

∗ These do not add up to 100%.

### 3.2 Participants, 2016

The participants (N = 262) of the study in 2016 comprised 10% of authorized veterinarians (N = 2633, the Registry of Veterinarians, Finnish Food Authority). Details of the 2016 study participants are reported in [27]: the majority were female (81.4%), and half (50.4%) had graduated within 10 years.

### 3.3 Exposure to zoonoses, 2009

All but two (99.3%) of the veterinarians who participated in the study in 2009 reported work-related contact with live animals, carcasses, or samples of animal origin; contacts with dogs, cats, cattle, and horses were common (Table 2). More than 90% reported having been exposed to zoonotic pathogens in their work, whereas 15.0% reported knowing that they had had a zoonosis (Table 2). Almost 80% reported having stuck themselves with a needle that had been in an animal (Table 2). Many, 85.0%, had been bitten, and 13.5% of them had been on sick leave because of a bite (Table 2). The biting animals were mainly those seen in small animal practice (dog, cat, rodents), but bite injuries caused by other animals (horse, cow, pig, sheep, bird) were also reported. Infected skin lesions were reported by 24.2% (Table 2).

**Table 2:** Exposure to different animal species and zoonotic pathogens as reported by veterinarians in Finland in the study in 2009 (N = 306)

	Yes			No			No answer		
	n	%	95% CI	n	%	95% CI	n	%	95% CI
**Exposure to any animal species**∗	304	99.3	97.6–99.8	2	0.7	0.2–2.4	0	0.0	0.0–1.2
Dog	294	96.1	93.3–97.7	11	3.6	2.0–6.3	1	0.3	0.1–1.8
Cat	292	95.4	92.5–97.3	13	4.2	2.5–7.1	1	0.3	0.1–1.8
Horse	250	81.7	77.0–85.6	55	18.0	14.1–22.7	1	0.3	0.1–1.8
Cattle	244	79.7	74.9–83.9	61	19.9	15.8–24.8	1	0.3	0.1–1.8
Rabbit	242	79.1	74.2–83.3	63	20.6	16.4–25.5	1	0.3	0.1–1.8
Swine	228	74.5	69.3–79.1	77	25.2	20.6–30.3	1	0.3	0.1–1.8
Small rodent	214	69.9	64.6–74.8	91	29.7	24.9–35.1	1	0.3	0.1–1.8
Sheep	204	66.7	61.2–71.7	101	33.0	28.0–38.5	1	0.3	0.1–1.8
Goat	146	47.7	42.2–53.3	159	52.0	46.4–57.5	1	0.3	0.1–1.8
Poultry	140	45.8	40.3–51.4	165	53.9	48.3–59.4	1	0.3	0.1–1.8
Cage bird	138	45.1	39.6–50.7	167	54.6	49.0–60.1	1	0.3	0.1–1.8
Reptile	125	40.9	35.5–46.4	180	58.8	53.2–64.2	1	0.3	0.1–1.8
Wild animal	95	31.0	26.1–36.4	210	68.6	63.2–73.6	1	0.3	0.1–1.8
Wild boar	84	27.5	22.8–32.7	221	72.2	67.0–76.9	1	0.3	0.1–1.8
Fish	68	22.2	17.9–27.2	237	77.5	72.4–81.8	1	0.3	0.1–1.8
Reindeer	66	21.6	17.3–26.5	239	78.1	73.1–82.4	1	0.3	0.1–1.8
Camelid	58	19.0	15.0–23.7	247	80.7	75.9–84.7	1	0.3	0.1–1.8
Fur animal	48	15.7	12.0–20.2	257	84.0	79.5–87.7	1	0.3	0.1–1.8
**Been exposed to zoonoses at work**	278	90.9	87.1–93.6	24	7.8	5.3–11.4	4	1.3	0.5–3.3
**Knew to have had a zoonosis**	46	15.0	11.5–19.5	234	76.5	71.4–80.9	26	8.5	5.9–12.2
**Has had infected skin lesion**	74	24.2	19.7–29.3	204	66.7	61.2–71.7	28	9.2	6.4–12.9
**Has had vesicular skin lesion**	15	4.9	3.0–7.9	268	87.6	83.4–90.8	23	7.5	5.1–11.0
**Has stuck themselves with a needle that has been in an animal**	241	78.8	73.8–83.0	43	14.1	10.6–18.4	22	7.2	4.8–10.6
**Has been bitten by an animal**	260	85.0	80.5–88.5	45	14.7	11.2–19.1	1	0.3	0.1–1.8
Has had infected animal bite†	146	56.2	50.1–62.1	110	42.3	36.5–48.4	4	1.5	0.6–3.9
Sick leave because of animal bite†	35	13.5	9.8–18.1	217	83.5	78.5–87.5	8	3.1	1.6–6.0
Hospital treatment because of animal bite†	5	1.9	0.8–4.4	244	93.9	90.2–96.2	11	4.2	2.4–7.4

CI, Confidence interval; n, Number of participants choosing each option.

∗ Includes exposure to live animal, carcass, or sample from animals.

† Of veterinarians who reported having been bitten by an animal (n = 260).

### 3.4 Self-evaluated knowledge of zoonoses, 2009 versus 2016

In the study in 2009, 8.2% of the participating veterinarians fully agreed with the claim “I have good knowledge of zoonoses and their prevention” (Table 3), while the proportion was 10.3% in 2016. In 2009, 89.5% selected one of the agreeing options (“slightly agree” or “agree” or “fully agree”), while the proportion was 85.9% in 2016. These differences were not statistically significant. The proportion of participating veterinarians selecting “slightly agree” decreased from 2009 to 2016 (50.0% vs. 28.6%; *p* = 0.001), while the proportion selecting “agree” increased (31.4% vs. 46.9%; *p* = 0.001).

**Table 3:** Self-assessed knowledge of zoonoses and their prevention by veterinarians in Finland: agreement with the claim “I have good knowledge of zoonoses and their prevention”

	2009 (N = 306)			2016 (N = 262)			Change in percentage unit	2009 vs*.* 2016	
	n	%	95% CI	n	%	95% CI		P-value	Corrected P-value
Fully disagree	3	1.0	0.3–2.8	2	0.8	0.2–2.7	-0.2	0.802	0.802
Disagree	0	0.0	0.0–1.2	0	0.0	0.0–1.5	0.0	N/A	
Slightly disagree	24	7.8	5.3–11.4	25	9.5	6.6–13.7	+1.7	0.471	0.538
Neither agree nor disagree	5	1.6	0.7–3.8	8	3.1	1.6–5.9	+1.5	0.234	0.374
Slightly agree∗	153	50.0	44.4–55.6	75	28.6	23.5–34.4	-21.4	<0.001	0.001
Agree∗	96	31.4	26.4–36.8	123	46.9	41.0–53.0	+15.5	<0.001	0.001
Fully agree	25	8.2	5.6–11.8	27	10.3	7.2–14.6	+2.1	0.387	0.516
No answer	0	0.0	0.0–1.2	2	0.8	0.2–2.7	+0.8	0.117	0.312
Fully agree, agree or slightly agree	274	89.5	85.6–92.5	225	85.9	81.1–89.6	-3.6	0.183	0.365

CI, confidence interval; N, total number of answers to this question; n, number of participants choosing each option; N/A, not applicable.

∗ Statistically significant at 5% level.

### 3.5 Protective practices, 2009

**Fig. 1:**
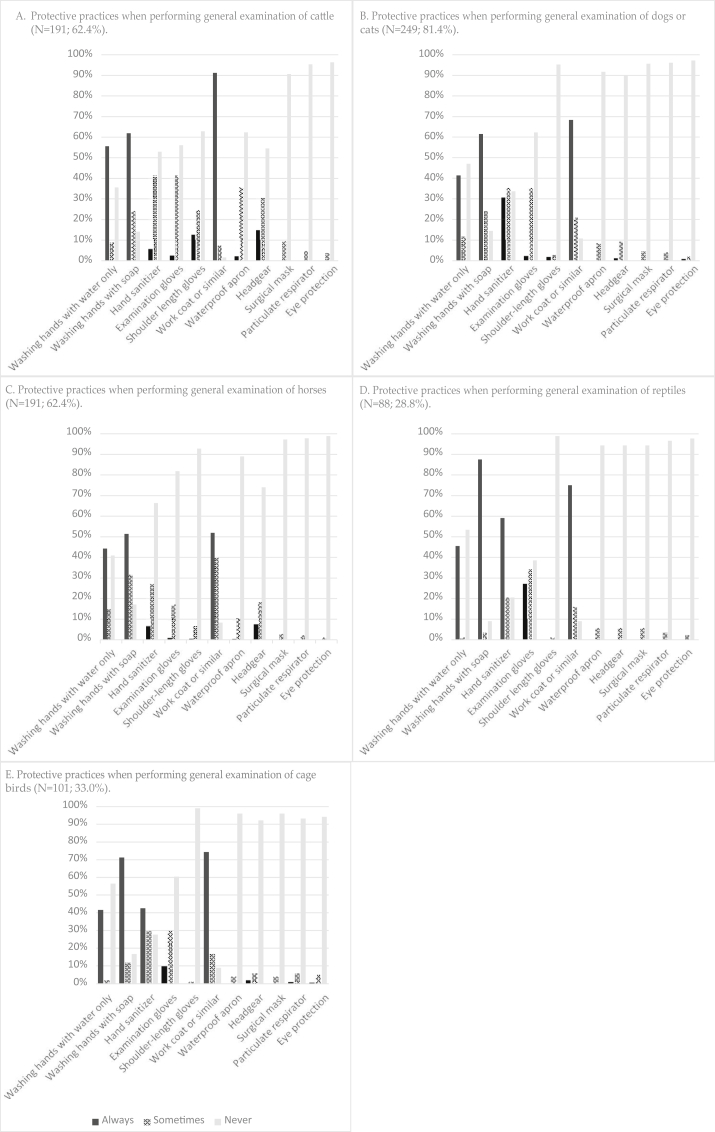
Protective practices in connection with general examination of different animal species as reported in 2009 by veterinarians authorized in Finland (N = 306).

The reported use of protective practices in connection with the specific veterinary procedures in the study in 2009 is summarized in Fig. 1, Table 4, and Supplementary Tables 1–12.

**Table 4:** Hand hygiene practices in connection with the examination of infected wounds of small animals and horses as reported by veterinarians in Finland: A) use of gloves in the study in 2009 and in the study in 2016 and B) hand wash and use of hand sanitizer and gloves in the study in 2009

A)	2009			2016			Change in percentage unit	2009 vs*.* 2016	
Use of examination gloves	n	%	95% CI	n	%	95% CI		P-value	Corrected P-value
**Small animal**∗	**N = 244**			**N = 179**					
Always	201	82.4	77.1−86.6	152	84.9	78.9−89.4	+2.5	0.494	0.593
Sometimes†	29	11.9	8.4−16.5	27	15.1	10.6−21.1	+3.2	0.338	0.506
Never‡	14	5.7	3.5−9.4	0	0.0	0.0−2.1	-5.7	0.001	0.007
**Horse**	**N = 162**			**N = 101**					
Always	110	67.9	60.4−74.6	80	79.2	70.3−86.0	+11.3	0.047	0.093
Sometimes†	35	21.6	16.0−28.6	20	19.8	13.2−28.6	-1.8	0.727	0.727
Never‡	17	10.5	6.7−16.2	1	1.0	0.2−5.4	-9.5	0.003	0.009

B)	Dog or cat (N = 244)			Horse (N = 162)			Dog or cat vs*.* horse	
	n	%	95% Cl	n	%	95 % Cl	P-value	Corrected P-value
**Washing hands with water only**								
Always	98	40.2	34.2–46.4	80	49.4	41.8–57.0	0.067	0.099
Sometimes	10	4.1	2.2–7.4	12	7.4	4.3–12.5	0.150	0.180
Never‡	136	55.7	49.5–61.8	70	43.2	35.8–50.9	0.014	0.023
**Washing hands with soap**								
Always‡	189	77.5	71.8–82.3	105	64.8	57.2–71.8	0.005	0.012
Sometimes‡	21	8.6	5.7–12.8	32	19.8	14.4–26.6	0.001	0.003
Never	34	13.9	10.1–18.8	25	15.4	10.7–21.8	0.674	0.674
**Hand sanitizer**								
Always‡	117	48.0	41.8–54.2	33	20.4	14.9–27.2	<0.001	0.001
Sometimes	54	22.1	17.4–27.7	40	24.7	18.7–31.9	0.543	0.592
Never‡	73	29.9	24.5–35.9	89	54.9	47.3–62.4	<0.001	0.001
**Use of examination gloves**								
Always‡	201	82.4	77.1–86.6	110	67.9	63.4–74.6	0.001	0.003
Sometimes‡	29	11.9	8.4–16.5	35	21.6	16.0–28.6	0.009	0.017
Never	14	5.7	3.4–9.4	17	10.5	6.7–16.2	0.074	0.099

CI, confidence interval; N, total number of answers to this question; n, number of participants choosing each option.

∗ In 2009, the questions specified “dog or cat”, and in 2016 “small animals”, which may include other small animals. The majority of small animals seen by veterinarians in Finland are dogs and cats.

† Includes often, sometimes and seldom in 2016 questionnaire answers.

‡ Statistically significance at 5% level.

In connection with general examination of cattle, 61.8% of the veterinarians reported to always wash their hands with soap and 91.1% to always wear a protective coat or similar (Fig. 1A; Supplementary Table 1). A protective coat was reportedly used by 68.3% for general examination of small animals and by 51.9% when examining horses (Fig. 1B and C; Supplementary Tables 2, 3). In connection with general examination of reptiles, 87.5% reported to always wash their hands with soap, 59.1% always use hand sanitizer, 27.3% always use gloves, and 75.0% always use a protective work coat or similar (Fig. 1D; Supplementary Table 4).

In connection with examination of a pig with erysipelas, 75.2% of the veterinarians reported always washing hands with soap, 18.4% always using hand sanitizer, and 26.4% always using gloves (Supplementary Table 5). When teat vesicles in cows were examined, 15.1% always used gloves (Supplementary Table 6). Of the 20 veterinarians who performed oral cavity examination on reindeer, 4 (20.0%) reported always wearing gloves in connection with this procedure (Supplementary Table 7). In connection with oral cavity examination of a dog or a cat, most (71.3%) reported always washing their hands with soap, 38.3% used hand sanitizer, and 28.8% used gloves (Supplementary Table 8). In connection with taking fecal samples from cattle, 76.2% reported washing hands always with soap, and more than 95% never used a surgical mask or particulate respirator (Supplementary Table 9). In connection with assisting cows in calving, more than 96% never used a surgical mask or particulate respirator (Supplementary Table 10). In connection with general examination of cage birds, a surgical mask or particulate respirator was never used by 96.0% and 93.1%, respectively (Fig. 1E; Supplementary Table 11). Furthermore, 94.1% never used eye protection in connection with examining cage birds, and 60.4% never used gloves. In connection with removal of dental calculus from dogs or cats, 82.1% reported always using gloves, 38.0% a surgical mask, 13.3% a particulate respirator, and 10.3% eye protection (Supplementary Table 12).

### 3.6 Protective practices, 2009 versus 2016

On examination of infected wounds in small animals, gloves were reportedly always used by 82.4% of the veterinarians in the study in 2009 and 84.9% in the study in 2016, and never used by 5.7% in 2009 and 0.0% in 2016 (Table 4A); the latter difference was statistically significant (*p* = 0.0072). For examination of infected wounds in horses, gloves were reportedly always used by 67.9% in the study in 2009 and 79.2% in 2016 [27], and never used by 10.5% in 2009 and 1.0% in 2016 [27] (Table 4A); the latter difference was statistically significant (*p* = 0.009). The proportions reportedly always using gloves, always using hand sanitizer, always washing hands with soap, and never washing hands with water only were significantly higher in connection with examining wounds in small animals than wounds in horses in the study in 2009 (Table 4B) (*p* = 0.0028, *p* = 0.0006, *p* = 0.0120, and *p* = 0.0233, respectively).

## 4 Discussion

The results we report from two questionnaire studies conducted among veterinarians in Finland add to the information on exposure of veterinarians to zoonoses and on the use of protective practices. A unique contribution are the detailed results related to specific veterinary procedures.

A substantial proportion (15% in 2009, 10% in 2016) of veterinarians of the country participated in the studies. The sample sizes were sufficient for overview, but due to different recruiting approaches and voluntary participation, the participants may not represent the profession well. For example, congress attendees may be a highly engaged professional group, and promotion in social media in 2016 may have caused overrepresentation of veterinarians following social media. Furthermore, veterinarians who were interested in zoonoses may have participated more likely, and potentially to both studies, which may have resulted in overestimation of the knowledge on zoonoses. The extent of participation in both studies was unknown but expected to be minor: possible non-independency would mean that the presented estimates are conservative. The age and gender distribution of the participants reflected the age-dependently increasing female dominance of the profession [30,31]. Awareness of zoonotic pathogens presenting reproductive risks [8] might differ by gender.

The participants of the 2009 study reported having had contact with a wide range of animal species, illustrating possibility to encounter a variety of zoonotic pathogens. It is also noteworthy that several participants reported immune system related diseases or immunosuppressive medication, which may predispose to infections and severe manifestations, and necessitate additional risk mitigation [5].

The exposure to zoonotic pathogens was common: more than 90% of the veterinarians participating in the 2009 study reported exposure. Every seventh (15.0%) veterinarian reported knowing that they had had a zoonosis, which is close to the proportions reported from North America (10.0–22%; [21,22,32]), but lower than those from Great Britain (44–64.5%; [20,33]), South Africa (63.6%; [34]), and Australia (44.9%; [19]). The proportion reportedly exposed to zoonotic pathogens was six times the proportion reporting to have had a zoonosis, which could indicate partial efficacy of the protective practices used.

The 78.8% of the veterinarians participating in the 2009 study who reported a needle stick incident (NSI) with a used needle may have become exposed to various pathogens, such as *Bartonella henselae* [35], hepatitis E virus [24], or mammalian bornaviruses [36,37]. NSIs were as common as in Portugal, with 78.5% of veterinarians reporting at least one NSI during their professional life [38]. Furthermore, 58.9% of veterinarians in studies in Australia [39] and 60% in Utah [21] reported at least one NSI during a year. Overall, NSI rate of 9.3–20 per 100 person-years has been estimated in veterinary practice [40], whereas in human health care, the rate is 1−5 NSIs per 1000 person-years [41]. More efforts to reduce NSIs in the veterinary profession are needed.

The majority (85.0%) of the veterinarians participating in the 2009 study had been bitten. The proportion corresponds with those reported from Canada, 63% of veterinarians having been bitten during the previous 5 years [32], and from the United States, 39.5% of the veterinary practitioners having had a skin-breaking bite within a year [21]. This is worrisome, as bite-transmissible zoonotic pathogens, such as *C. canimorsus* and rabies virus, can be life-threatening.

The two snapshots of proportions of veterinarians self-reportedly having good knowledge of zoonoses were similar (Table 3). The proportions (8.2% in 2009, 10.3% in 2016) fully agreeing with the claim “I have good knowledge of zoonoses” were lower than in a study in Australia, with 41.5% of veterinarians reporting a high level of knowledge of zoonoses [19]. Education about zoonoses and their relevance for occupational and public health should be increased during the whole professional life [22]. A prospective cohort study could be useful for identifying patterns in the development of knowledge but needs to take account the Hawthorne effect [42]: participation can have an improving effect itself. Further studies could also map the information sources veterinarians use, and investigate the compliance with occupational health and safety legislation, to yield relevant data for action.

It should be emphasized that both questionnaire studies were planned and performed before the national veterinary hygiene guideline [16] was published in 2019. The questions were not designed to investigate how specific guidelines or legislation were followed. According to the previous and current American Veterinary Standard Precautions [14,43], the former existing at the time of the studies, and also the recent national veterinary hygiene guideline [16], disposable gloves and protective outerwear should be worn when in contact with excreta, bodily fluids, and non-intact skin, as well as in dental and obstetric procedures. The guideline [16] also advises to use gloves when contact with mucous membranes is anticipated. Based on our results, there is a need to improve enforcement of these guidelines. Lapses in hand hygiene are worrying and may also enable the spread of resistant bacteria and human pathogens. The practices appeared slightly improved in 2016 in comparison with 2009 and may have further improved because of the COVID-19-related recommendations.

Protective outerwear should always be worn when attending to animals [14,16]. Similarly to previous questionnaire results from the United States [17], this appeared to be quite well followed in 2009 during cattle contact but less so with small animals and poorly with horses. Findings in line with these have been reported from the Great Britain, with 68.3% of veterinarians not using protective outerwear when in contact with small animals [20], and from Finland, based on questions somewhat differing from the 2009 study, among veterinarians in ambulatory livestock and equine practice [27].

Protection from fecal-oral pathogens has gained importance with an increasing incidence of *Cryptosporidium parvum* in cattle and humans [44]. Practices in connection with reptiles, a common *Salmonella* reservoir, also need improvement. Furthermore, not using gloves in swine contact has been associated with a higher risk of hepatitis E virus and *Ascaris suum* seropositivity [45]. Additionally, poor use of gloves may increase the risk of the cutaneous transmission of pathogens, including poxviruses [46,47] and fungi [19,20,32,34]. Further studies should also look into the types of gloves used.

Our results indicate a particular need for improving protection from pathogens transmissible via droplets, aerosols, and air. The Veterinary Standard Precautions [14,16] advise using facial protection when performing dental or obstetrical procedures. A face shield or eye protection with a surgical mask mostly suffices to protect from droplets, but particulate respirators are recommended to protect from airborne pathogens [14] such as *Coxiella burnetii* from ruminants, MRSA from swine, and *Chlamydophila psittaci* from birds.

Veterinarians should set an example in protective practices. For many pathogens, there is no full understanding of how much each protective practice contributes to decreasing risk. General, applicable check lists and guidance for the risk assessment regarding health and safety of workers are available [48,49].

Protective practices need to be adjusted to local situation. Despite the relatively good general zoonosis situation in Finland [50], infections with some endemic zoonotic pathogens such as *T. gondii* are common [8]. Veterinarians are also a risk group, and could be sentinels, for emerging pathogens, such as SARS-CoV-2 [12], zoonotic influenza, and vector-borne pathogens (reviewed by [5]).

In conclusion, majority of the veterinarians reported having been occupationally exposed to zoonotic pathogens, and NSIs and bite incidents were common, while it was evident that protective practices should be improved. It is always prudent to emphasize awareness, good work culture and processes, hygiene measures, and personal protective equipment, when in contact with animals. In addition to guidelines and education, One Health collaboration between veterinary professionals, medical doctors and occupational health care professionals is crucial in taking care of the health of veterinarians.

## Conflicts of interest

PMK is currently affiliated to MSD Animal Health. The studies were completed before the affiliation change, and MSD Animal Health has had no influence on the content of this article.

No other conflicts of interest.
