# Supplementary material for: Veterinarians as a Risk Group for Zoonoses: Exposure, Knowledge and Protective Practices in Finland
Source: Saf Health Work. 2021 Nov 9;13(1):78–85. doi: 10.1016/j.shaw.2021.10.008 (PMC9346934; doi:10.1016/j.shaw.2021.10.008)
Supplement: Multimedia component 1 [file mmc1.pdf]

Supplementary material

Supplementary Table 1. Protective practices in connection with bovine general examination as reported in 2009 by veterinarians in Finland (N=191)

Supplementary Table 2. Protective practices in connection with general examination of dogs and cats as reported in 2009 by veterinarians in Finland (N=249)

Supplementary Table 3. Protective practices in connection with general examination of horses as reported in 2009 by veterinarians in Finland (N=181)

Supplementary Table 4. Protective practices in connection with general examination of reptiles as reported in 2009 by veterinarians in Finland (N=88)

Supplementary Table 5. Protective practices in connection with the examination of swine with erysipelas as reported in 2009 by veterinarians in Finland (N=125)

Supplementary Table 6. Protective practices in connection with the examination of teat vesicles in cows as reported in 2009 by veterinarians in Finland (N=159)

Supplementary Table 7. Protective practices in connection with the examination of reindeer´s oral cavity as reported in 2009 by veterinarians in Finland (N=20)

Supplementary Table 8. Protective practices in connection with investigation of the oral cavity of dog or cat as reported in 2009 by veterinarians in Finland (N=240)

Supplementary Table 9. Protective practices when taking fecal samples from cattle as reported in 2009 by veterinarians in Finland (N=172)

Supplementary Table 10. Protective practices in connection with calving assistance as reported in 2009 by veterinarians in Finland (N=183)

Supplementary Table 11. Protective practices in connection with general examination of cage birds as reported in 2009 by veterinarians in Finland (N=101)

Supplementary Table 12. Protective practices in connection with the removal of dental calculus from dogs or cats as reported in 2009 by veterinarians in Finland (N=234)

Supplementary Table 1. Protective practices in connection with bovine general examination as reported in 2009 by veterinarians in Finland (N=191)

	Always			Sometimes			Never		
	n	%	95 % CI	n	%	95% CI	n	%	95% CI
**Washing hands with water only**	106	55.5	48.4–62.4	17	8.9	5.6–13.8	68	35.6	29.2–42.6
**Washing hands with soap**	118	61.8	54.7–68.4	46	24.1	18.6–30.6	27	14.1	9.9–19.8
**Hand sanitizer**	11	5.8	3.3–10.0	79	41.4	34.6–48.5	101	52.9	45.8–59.8
**Examination gloves**	5	2.6	1.1–6.0	79	41.4	34.6–48.5	107	56.0	48.9–62.9
**Shoulder-length gloves**	24	12.6	8.6–18.0	47	24.6	19.0–31.2	120	62.8	55.8–69.4
**Work coat or similar**	174	91.1	86.2–94.4	14	7.3	4.4–11.9	3	1.6	0.5–4.5
**Waterproof apron**	4	2.1	0.8–5.3	68	35.6	29.2–42.6	119	62.3	55.3–68.9
**Headgear**	29	15.2	10.8–21.0	58	30.4	24.3–37.2	104	54.5	47.4–61.4
**Surgical mask**	0	0.0	0.0–2.0	18	9.4	6.0–14.4	173	90.6	85.6–94.0
**Particulate respirator**	0	0.0	0.0–2.0	9	4.7	2.5–8.7	182	95.3	91.3–97.5
**Eye protection**	0	0.0	0.0–2.0	7	3.7	1.8–7.4	184	96.3	92.6–98.2

CI, Confidence interval; n, number of participants choosing each option.

Supplementary Table 2. Protective practices in connection with general examination of dogs and cats as reported in 2009 by veterinarians in Finland (N=249)

	Always			Sometimes			Never		
	n	%	95 % CI	n	%	95% CI	n	%	95% CI
**Washing hands with water only**	103	41.4	35.4–47.6	29	11.7	8.2–16.2	117	47.0	40.9–53.2
**Washing hands with soap**	153	61.5	55.3–67.3	60	24.1	19.2–29.8	36	14.5	10.6–19.4
**Hand sanitizer**	77	30.9	25.5–36.9	88	35.3	29.7–41.5	84	33.7	28.2–39.8
**Examination gloves**	6	2.4	1.1–5.2	88	35.3	29.7–41.5	155	62.3	56.1–68.0
**Shoulder-length gloves**	5	2.0	0.9–4.6	7	2.8	1.4–5.7	237	95.2	91.8–97.2
**Work coat or similar**	170	68.3	62.3–73.7	52	20.9	16.3–26.4	27	10.8	7.6–15.3
**Waterproof apron**	0	0.0	0.0–1.5	21	8.4	5.6–12.6	228	91.6	87.5–94.4
**Headgear**	3	1.2	0.4–3.5	23	9.2	6.2–13.5	223	89.6	85.1–92.8
**Surgical mask**	0	0.0	0.0–1.5	11	4.4	2.5–7.7	238	95.6	92.3–97.5
**Particulate respirator**	0	0.0	0.0–1.5	10	4.0	2.2–7.2	239	96.0	92.8–97.8
**Eye protection**	2	0.8	0.2–2.9	5	2.0	0.9–4.6	242	97.2	94.3–98.6

CI, Confidence interval; n, number of participants choosing each option.

Supplementary Table 3. Protective practices in connection with general examination of horses as reported in 2009 by veterinarians in Finland (N=181)

	Always			Sometimes			Never		
	n	%	95 % CI	n	%	95% CI	n	%	95% CI
**Washing hands with water only**	80	44.2	37.2–51.5	27	14.9	10.5–20.8	74	40.9	34.0–48.2
**Washing hands with soap**	93	51.4	44.2–58.6	57	31.5	25.2–38.6	31	17.1	12.3–23.3
**Hand sanitizer**	12	6.6	3.8–11.2	49	27.1	21.1–34.0	120	66.3	59.1–72.8
**Examination gloves**	2	1.1	0.3–3.9	31	17.1	12.3–23.3	148	81.8	75.5–86.7
**Shoulder-length gloves**	1	0.5	0.1–3.1	12	6.6	3.8–11.2	168	92.8	88.1–95.8
**Work coat or similar**	94	51.9	44.7–59.1	72	39.8	32.9–47.1	15	8.3	5.1–13.2
**Waterproof apron**	1	0.5	0.1–3.1	19	10.5	6.8–15.8	161	89.0	83.6–92.7
**Headgear**	14	7.7	4.7–12.6	33	18.2	13.3–24.5	134	74.0	67.2–79.9
**Surgical mask**	0	0.0	0.0–2.1	5	2.8	1.2–6.3	176	97.2	93.7–98.8
**Particulate respirator**	0	0.0	0.0–2.1	4	2.2	0.9–5.5	177	97.8	94.5–99.1
**Eye protection**	0	0.0	0.0–2.1	2	1.1	0.3–3.9	179	98.9	96.1–99.7

CI, Confidence interval; n, number of participants choosing each option.

Supplementary Table 4. Protective practices in connection with general examination of reptiles as reported in 2009 by veterinarians in Finland (N=88)

	Always			Sometimes			Never		
	n	%	95 % CI	n	%	95% CI	n	%	95% CI
**Washing hands with water only**	40	45.5	35.5–55.8	1	1.1	0.2–6.2	47	53.4	43.1–63.5
**Washing hands with soap**	77	87.5	79.0–92.9	3	3.4	1.2–9.6	8	9.1	4.7–16.9
**Hand sanitizer**	52	59.1	48.7–68.8	18	20.5	13.4–30.0	18	20.5	13.4–30.0
**Examination gloves**	24	27.3	19.1–37.4	30	34.1	25.0–44.5	34	38.6	29.1–49.1
**Shoulder-length gloves**	0	0.0	0.0–4.2	1	1.1	0.2–6.2	87	98.9	93.8–99.8
**Work coat or similar**	66	75.0	65.0–82.9	14	15.9	9.7–25.0	8	9.1	4.7–16.9
**Waterproof apron**	0	0.0	0.0–4.2	5	5.7	2.5–12.6	83	94.3	87.4–97.6
**Headgear**	0	0.0	0.0–4.2	5	5.7	2.5–12.6	83	94.3	87.4–97.6
**Surgical mask**	0	0.0	0.0–4.2	5	5.7	2.5–12.6	83	94.3	87.4–97.6
**Particulate respirator**	0	0.0	0.0–4.2	3	3.4	1.2–9.6	85	96.6	90.5–98.8
**Eye protection**	0	0.0	0.0–4.2	2	2.3	0.6–7.9	86	97.7	92.1–99.4

CI, Confidence interval; n, number of participants choosing each option.

Supplementary Table 5. Protective practices in connection with the examination of swine with erysipelas as reported in 2009 by veterinarians in Finland (N=125)

	Always			Sometimes			Never		
	n	%	95 % CI	n	%	95% CI	n	%	95% CI
**Washing hands with water only**	55	44.0	35.6–52.8	4	3.2	1.3–7.9	66	52.8	44.1–61.3
**Washing hands with soap**	94	75.2	67.0–81.9	13	10.4	6.2–17.0	18	14.4	9.3–21.6
**Hand sanitizer**	23	18.4	12.6–26.1	29	23.2	16.7–31.3	73	58.4	49.6–66.7
**Examination gloves**	33	26.4	19.5–34.8	27	21.6	15.3–29.6	65	52.0	43.3–60.6
**Shoulder-length gloves**	1	0.8	0.1–4.4	2	1.6	0.4–5.7	122	97.6	93.2–99.2
**Work coat or similar**	123	98.4	94.4–99.6	1	0.8	0.1–4.4	1	0.8	0.1–4.4
**Waterproof apron**	2	1.6	0.4–5.7	7	5.6	2.7–11.1	116	92.8	86.9–96.2
**Headgear**	37	29.6	22.3–38.1	21	16.8	11.3–24.3	67	53.6	44.9–62.1
**Surgical mask**	1	0.8	0.1–4.4	1	0.8	0.1–4.4	123	98.4	94.4–99.6
**Particulate respirator**	4	3.2	1.3–7.9	10	8.0	4.4–14.1	111	88.8	82.1–93.2
**Eye protection**	2	1.6	0.4–5.7	2	1.6	0.4–5.7	121	96.8	92.1–98.8

CI, Confidence interval; n, number of participants choosing each option.

Supplementary Table 6. Protective practices in connection with the examination of teat vesicles in cows as reported in 2009 by veterinarians in Finland (N=159)

	Always			Sometimes			Never		
	n	%	95 % CI	n	%	95% CI	n	%	95% CI
**Washing hands with water only**	83	52.2	44.5–59.8	7	4.4	2.2–8.8	69	43.4	35.9–51.2
**Washing hands with soap**	119	74.8	67.6–81.0	17	10.7	6.8–16.5	23	14.5	9.8–20.8
**Hand sanitizer**	23	14.5	9.8–20.8	45	28.3	21.9–35.8	91	57.2	49.5–64.7
**Examination gloves**	24	15.1	10.4–21.5	55	34.6	27.6–42.3	80	50.3	42.6–58.0
**Shoulder-length gloves**	0	0.0	0.0–2.4	8	5.0	2.6–9.6	151	95.0	90.4–97.4
**Work coat or similar**	144	90.6	85.0–94.2	7	4.4	2.2–8.8	8	5.0	2.6–9.6
**Waterproof apron**	5	3.1	1.4–7.2	22	13.8	9.3–20.1	132	83.0	76.4–88.1
**Headgear**	23	14.5	9.8–20.8	33	20.8	15.2–27.7	103	64.8	57.1–71.8
**Surgical mask**	0	0.0	0.0–2.4	5	3.1	1.4–7.2	154	96.9	92.9–98.7
**Particulate respirator**	0	0.0	0.0–2.4	4	2.5	1.0–6.3	155	97.5	93.7–99.0
**Eye protection**	1	0.6	0.1–3.5	5	3.1	1.4–7.2	153	96.2	92.0–98.3

CI, Confidence interval; n, number of participants choosing each option.

Supplementary Table 7. Protective practices in connection with the examination of reindeer´s oral cavity as reported in 2009 by veterinarians in Finland (N=20)

	Always			Sometimes			Never		
	n	%	95 % CI	n	%	95% CI	n[a]	%	95% CI
**Washing hands with water only**	9	45.0	25.8–65.8	4	20.0	8.1–41.6	7	35.0	18.1–56.7
**Washing hands with soap**	14	70.0	48.1–85.5	5	25.0	11.2–46.9	1	5.0	0.9–23.6
**Hand sanitizer**	3	15.0	5.2–36.0	8	40.0	21.9–61.3	9	45.0	25.8–65.8
**Examination gloves**	4	20.0	8.1–41.6	10	50.0	29.9–70.1	6	30.0	14.6–51.9
**Shoulder-length gloves**	0	0.0	0.0–16.1	0	0.0	0.0–16.1	20	100.0	83.9–100.0
**Work coat or similar**	15	75.0	53.1–88.8	2	10.0	2.8–30.1	3	15.0	5.2–36.0
**Waterproof apron**	0	0.0	0.0–16.1	0	0.0	0.0–16.1	20	100.0	83.9–100.0
**Headgear**	3	15.0	5.2–36.0	2	10.0	2.8–30.1	15	75.0	53.1–88.8
**Surgical mask**	0	0.0	0.0–16.1	0	0.0	0.0–16.1	20	100.0	83.9–100.0
**Particulate respirator**	0	0.0	0.0–16.1	0	0.0	0.0–16.1	20	100.0	83.9–100.0
**Eye protection**	0	0.0	0.0–16.1	0	0.0	0.0–16.1	20	100.0	83.9–100.0

CI, Confidence interval; n, number of participants choosing each option.

Supplementary Table 8. Protective practices in connection with investigation of the oral cavity of dog or cat as reported in 2009 by veterinarians in Finland (N=240)

	Always			Sometimes			Never		
	n	%	95 % CI	n	%	95% CI	n	%	95% CI
**Washing hands with water only**	91	37.9	32.0–44.2	11	4.6	2.6–8.0	138	57.5	51.2–63.6
**Washing hands with soap**	171	71.3	65.2–76.6	27	11.3	7.9–15.9	42	17.5	13.2–22.8
**Hand sanitizer**	92	38.3	32.4–44.6	61	25.4	20.3–31.3	87	36.3	30.4–42.5
**Examination gloves**	69	28.8	23.4–34.8	120	50.0	43.7–56.3	51	21.3	16.6–26.9
**Shoulder-length gloves**	2	0.8	0.2–3.0	4	1.7	0.7–4.2	234	97.5	94.7–98.9
**Work coat or similar**	172	71.7	65.7–77.0	39	16.3	12.1–21.4	29	12.1	8.6–16.8
**Waterproof apron**	1	0.4	0.1–2.3	20	8.3	5.5–12.5	219	91.3	87.0–94.2
**Headgear**	4	1.7	0.7–4.2	20	8.3	5.5–12.5	216	90.0	85.6–93.2
**Surgical mask**	3	1.3	0.4–3.6	17	7.1	4.5–11.1	220	91.7	87.5–94.5
**Particulate respirator**	1	0.4	0.1–2.3	9	3.8	2.0–7.0	230	95.8	92.5–97.7
**Eye protection**	2	0.8	0.2–3.0	8	3.3	1.7–6.4	230	95.8	92.5–97.7

CI, Confidence interval; n, number of participants choosing each option.

Supplementary Table 9. Protective practices when taking fecal samples from cattle as reported in 2009 by veterinarians in Finland (N=172)

	Always			Sometimes			Never		
	n	%	95 % CI	n	%	95% CI	n	%	95% CI
**Washing hands with water only**	86	50.0	42.6–57.4	7	4.1	2.0–8.2	79	45.9	38.7–53.4
**Washing hands with soap**	131	76.2	69.3–81.9	15	8.7	5.4–13.9	26	15.1	10.5–21.2
**Hand sanitizer**	21	12.2	8.1–17.9	48	27.9	21.7–35.0	103	59.9	52.4–66.9
**Examination gloves**	56	32.6	26.0–39.9	26	15.1	10.5–21.2	90	52.3	44.9–59.7
**Shoulder-length gloves**	114	66.3	58.9–72.9	18	10.5	6.7–15.9	40	23.3	17.6–30.1
**Work coat or similar**	151	87.8	82.1–91.9	6	3.5	1.6–7.4	15	8.7	5.4–13.9
**Waterproof apron**	23	13.4	9.1–19.3	41	23.8	18.1–30.7	108	62.8	55.4–69.7
**Headgear**	27	15.7	11.0–21.9	35	20.4	15.0–27.0	110	64.0	56.6–70.8
**Surgical mask**	0	0.0	0.0–2.2	7	4.1	2.0–8.2	165	95.9	91.8–98.0
**Particulate respirator**	0	0.0	0.0–2.2	5	2.9	1.3–6.6	167	97.1	93.4–98.8
**Eye protection**	1	0.6	0.1–3.2	5	2.9	1.3–6.6	166	96.5	92.6–98.4

CI, Confidence interval; n, number of participants choosing each option.

Supplementary Table 10. Protective practices in connection with calving assistance as reported in 2009 by veterinarians in Finland (N=183)

	Always			Sometimes			Never		
	n	%	95 % CI	n	%	95% CI	n	%	95% CI
**Washing hands with water only**	96	52.5	45.3–59.6	8	4.4	2.2–8.4	79	43.2	36.2–50.4
**Washing hands with soap**	148	80.9	74.6–85.9	11	6.0	3.4–10.4	24	13.1	9.0–18.8
**Hand sanitizer**	42	23.0	17.5–29.6	50	27.3	21.4–34.2	91	49.7	42.6–56.9
**Examination gloves**	13	7.1	4.2–11.8	39	21.3	16.0–27.8	131	71.6	64.7–77.6
**Shoulder-length gloves**	18	9.8	6.3–15.0	82	44.8	37.8–52.1	83	45.4	38.3–52.6
**Work coat or similar**	100	54.6	47.4–61.7	17	9.3	5.9–14.4	66	36.1	29.5–43.2
**Waterproof apron**	145	79.2	72.8–84.5	23	12.6	8.5–18.2	15	8.2	5.0–13.1
**Headgear**	32	17.5	12.7–23.6	41	22.4	17.0–29.0	110	60.1	52.9–66.9
**Surgical mask**	0	0.0	0.0–2.1	6	3.3	1.5–7.0	177	96.7	93.0–98.5
**Particulate respirator**	1	0.6	0.1–3.0	4	2.2	0.9–5.5	178	97.3	93.8–98.8
**Eye protection**	1	0.6	0.1–3.0	5	2.7	1.2–6.2	177	96.7	93.0–98.5

CI, Confidence interval; n, number of participants choosing each option.

Supplementary Table 11. Protective practices in connection with general examination of cage birds as reported in 2009 by veterinarians in Finland (N=101)

	Always			Sometimes			Never		
	n	%	95 % CI	n	%	95% CI	n	%	95% CI
**Washing hands with water only**	42	41.6	32.5–51.3	2	2.0	0.5–6.9	57	56.4	46.7–65.7
**Washing hands with soap**	72	71.3	61.8–79.2	12	11.9	6.9–19.6	17	16.8	10.8–25.3
**Hand sanitizer**	43	42.6	33.4–52.3	30	29.7	21.7–39.2	28	27.7	19.9–37.2
**Examination gloves**	10	9.9	5.5–17.3	30	29.7	21.7–39.2	61	60.4	50.7–69.4
**Shoulder-length gloves**	0	0.0	0.0–3.7	1	1.0	0.2–5.4	100	99.0	94.6–99.8
**Work coat or similar**	75	74.3	65.0–81.8	17	16.8	10.8–25.3	9	8.9	4.8–16.1
**Waterproof apron**	0	0.0	0.0–3.7	4	4.0	1.6–9.7	97	96.0	90.3–98.5
**Headgear**	2	2.0	0.5–6.9	6	5.9	2.8–12.4	93	92.1	85.1–95.9
**Surgical mask**	0	0.0	0.0–3.7	4	4.0	1.6–9.7	97	96.0	90.3–98.5
**Particulate respirator**	1	1.0	0.2–5.4	6	5.9	2.8–12.4	94	93.1	86.4–96.6
**Eye protection**	1	1.0	0.2–5.4	5	5.0	2.1–11.1	95	94.1	87.6–97.3

CI, Confidence interval; n, number of participants choosing each option.

Supplementary Table 12. Protective practices in connection with the removal of dental calculus from dogs or cats as reported in 2009 by veterinarians in Finland (N=234)

	Always			Sometimes			Never		
	n	%	95 % CI	n	%	95% CI	n	%	95% CI
**Washing hands with water only**	93	39.7	33.7–46.1	9	3.9	2.0–7.2	132	56.4	50.0–62.6
**Washing hands with soap**	175	74.8	68.9–79.9	21	9.0	5.9–13.3	38	16.2	12.1–21.5
**Hand sanitizer**	94	40.2	34.1–46.6	59	25.2	20.1–31.2	81	34.6	28.8–40.9
**Examination gloves**	192	82.1	76.6–86.4	21	9.0	5.9–13.3	21	9.0	5.9–13.3
**Shoulder-length gloves**	2	0.9	0.2–3.1	4	1.7	0.7–4.3	228	97.4	94.5–98.8
**Work coat or similar**	172	73.5	67.5–78.8	29	12.4	8.8–17.2	33	14.1	10.2–19.1
**Waterproof apron**	12	5.1	3.0–8.8	24	10.3	7.0–14.8	198	84.6	79.4–88.7
**Headgear**	24	10.3	7.0–14.8	36	15.4	11.3–20.6	174	74.4	68.4–79.5
**Surgical mask**	89	38.0	32.1–44.4	31	13.3	9.5–18.2	114	48.7	42.4–55.1
**Particulate respirator**	31	13.3	9.5–18.2	13	5.6	3.3–9.3	190	81.2	75.7–85.7
**Eye protection**	24	10.3	7.0–14.8	36	15.4	11.3–20.6	174	74.4	68.4–79.5

CI, Confidence interval; n, number of participants choosing each option.
